# Transforming Intractable Policy Conflicts: A Qualitative Study Examining the Novel Application of Facilitated Discourse (Track Two Diplomacy) to Community Water Fluoridation in Calgary, Canada

**DOI:** 10.3390/ijerph20146402

**Published:** 2023-07-19

**Authors:** Aleem Bharwani, Jessica Van Dyke, Cristina Santamaria-Plaza, Julia Palmiano Federer, Peter Jones

**Affiliations:** 1 UCalgary Pluralism Initiative and O’Brien Institute for Public Health, University of Calgary, Calgary, AB T2N 4N1, Canada; 2 Ward of the 21st Century, University of Calgary, Calgary, AB T2N 4N1, Canada; 3 Department of Community Health Sciences, University of Calgary, Calgary, AB T2N 4N1, Canada; 4 Ottawa Dialogue, University of Ottawa, Ottawa, ON K1N 5Y3, Canada

**Keywords:** track two diplomacy, intractable conflicts, pluralism, community water fluoridation, polarization, public policy, qualitative, facilitated discourse, quiet diplomacy

## Abstract

Governments face challenges in resolving complex health and social policy conflicts, such as the community water fluoridation (CWF) impasse in Calgary. Track Two diplomacy, informal dialogues facilitated by an impartial third party, is proposed to address these issues amid epistemic conflict and declining public trust in fellow citizens, science, and government. This study examined Track Two diplomacy’s application in Calgary’s CWF policy conflict. Collaborating with policymakers and community partners, the research team explored a Track Two–CWF process and conducted 21 semi-structured interviews with policymakers, scholars, practitioners, observers, and civil society representatives. Data interpretation explored contextual factors, conflict transformation potential, and design features for a Track Two process. A conflict map revealed factors contributing to impasse: the polarizing nature of a binary policy question on fluoridation; disciplinary silos; failed public engagement; societal populism; societal lack of disposition to dialogue; individual factors (adverse impact of conflict on stakeholders, adherence to extreme positions, issue fatigue, apathy, and lack of humility); together with policy-making factors (perceived lack of leadership, lack of forum to dialogue, polarization and silos). Participants suggested reframing the issue as nonbinary, involving a skilled facilitator, convening academics, and considering multiple dialogue tracks for a Track Two process. The first theory of change would focus on personal attitudes, relationships, and culture. Participants expressed cautious optimism about Track Two diplomacy’s potential. Track Two diplomacy offers a promising approach to reframe intractable public health policy conflicts by moving stakeholders from adversarial positions to jointly assessing and solving problems. Further empirical evidence is needed to test the suggested process.

## 1. Introduction

Governments are increasingly confronted by intractable policy conflicts [1,2]. Particularly in the context of complex health and social issues, policy-making often reaches an impasse when faced with a “wicked” problem, which is characterized by divergent values, perspectives, and uncertainties surrounding causal relationships and policy impacts [3].

Different “truths” emerge across disciplines, sectors, and identities. Distinct identity groups define important paradigms, problematics, and concepts differently [4], including proponents and opponents of policies who privilege different disciplinary evidence or disagree ideologically on the role of public health, including the legitimacy of universal interventions for collective benefit. Credibility of knowledge claims is frequently questioned [5], and disciplinary adherence is often reinforced by a lack of shared understanding across disciplines [6]. Hindenlang et al. describe an example where within-group rigidity can become so deep that “scientific facts, value judgements, and intention” can be conflated [7].

Amidst an increasingly complex, populist world marked by distrust for science and public institutions, we increasingly need mechanisms to facilitate constructive contact across axes of difference. Trust in science has declined [5] whether related to increased data falsification and fabrication [8]—or to the public’s discomfort with uncertainty, where science is expected to provide indisputable truths and when it presents uncertainties “people wonder how little difference there is between politics and science” [5]. That science and politics are coupled is not, necessarily, the source of public unease. Rather, it is the lack of reflexiveness within these institutions that creates apprehension, resulting in public skepticism towards evidence [9].

These factors contribute to failed public health policies and exacerbate intergroup conflicts. Such issues are hallmarks of the community water fluoridation (CWF) policy impasse in Calgary, where policymaker, community, and scholar support and opposition present formidable challenges for resolution in a city that has just completed its seventh plebiscite on the issue in 60 years [10,11].

As part of a knowledge synthesis conducted by the University of Calgary [10], authors witnessed and heard about vicious accusations, attacks, and mischaracterizations of evidence. Opponents and proponents often privileged certain evidence and rejected other evidence often assuming malicious intent by the other side. Tight and recurring referenda and uncivil discourse defines the CWF conflict in Calgary as well as in other North American cities [12], and, as the pandemic has demonstrated, has come to shape any number of public health issues from vaccinations to racism [13].

Public health policymakers lack evidence-based approaches to resolve such intractable conflicts. Track Two diplomacy can offer potential solutions. Rather than reduce the debate to one of unequivocal positions, Track Two diplomacy creates the conditions of possibility—where multiple views hold weight and resolutions are found in transforming dichotomous thinking into a more realistic, if not more complicated, whole.

Track Two diplomacy is an informal, unofficial dialogue between conflicting parties, facilitated by an impartial third party [14]. This method of facilitated discourse has contributed to international conflict resolution [15] and is now being considered in public health conflicts [11]. By adapting Track Two diplomacy for public health policy conflicts, such as Calgary’s CWF debate, we intended to examine this innovative approach to resolving intractable conflicts and contribute to the pursuit of long-term, durable solutions.

Track Two diplomacy began in the 1960s when social scientists who were concerned that traditional methods of diplomacy and models of international relations did not provide sufficient scope to manage intractable, identity-based conflicts, began searching for alternate ways to approach such conflicts. The method they developed, and which has continued to develop since, is grounded in social psychology and places substantial importance on understanding and addressing the emotional and psychological aspects of the conflict. This method creates conditions to dive beyond superficial rationalization and in doing so stresses the importance of facilitated dialogue as a means of shifting the discourse between groups in conflict from one of zero-sum bargaining towards a “problem-solving” mode of interaction. The key to this is the observation that groups in conflict, if brought together in an unstructured way, will quickly fall into positional advocacy, which stands very little chance of success in an atmosphere where the differences are so ingrained that neither side is inclined to find ways to “give” anything. Instead, they seek to defend their positions and maximise their potential gains.

The proponents of what would become Track Two argued that a skilled facilitator should have the job of gently nudging the protagonists into a reflective, analytical mode of interaction which would have the goal of their coming to see the issue between them not as a difference to be bargained over, but as a problem which they jointly share. They would then move on to a stage whereby they would engage in a joint analysis of the “problem,” leading to a further stage where they would jointly search for ways to “solve” the problem on the basis of “sum–sum” approaches. The field has continued to develop, both in theory and as case studies have added to our understanding of its potential [14,16].

## 2. Methods

### 2.1. Aim and Design

To better understand whether and how facilitated discourse could transform intractable public health policy issues in Calgary, Canada, the research team partnered with a policymaker (City of Calgary), and community partners (two city-wide, generalist, umbrella civil society organizations) to explore and develop a Track Two–CWF process. We sought to determine whether Track Two diplomacy methods could contribute to public health policy impasses, namely CWF, in pursuit of a more durable, long-term solution. This study was approved by the University of Calgary Health Research Ethics Board (REB21-1139). We followed the Consolidated Criteria for Reporting Qualitative Research (COREQ) checklist to ensure rigorous and transparent reporting of our qualitative research methods.

The research team comprised two Track Two diplomacy experts, one municipal governance expert, one community water fluoridation scholar, one graduate student in public health, and one scholar with expertise in applying quiet diplomacy to the community water fluoridation issue.

### 2.2. Participants

We convened an academic–policymaker–community partner committee comprising the research team, our partner policymaker from the city, and community partner representatives. The committee jointly reviewed and provided feedback on the interview guide and identified an initial sample of participants in each of the predetermined categories listed below to obtain an initial invite list after which we proceeded to recruit through snowball sampling with the goal of having at least two participants in each category. We invited 44 participants by email of which 1 declined, 23 agreed, and 21 were scheduled. We stopped recruiting once we reached data saturation and no new themes were emerging.

### 2.3. Data Collection

AB, the principal investigator trained in qualitative research, conducted 21 ~1 h semi-structured interviews with 21 participants over Zoom between August 2021 and January 2022; a graduate student CSP, with qualitative research experience, was present for all interviews. The 21 participants comprised elected city councillors (5), senior city administrators (2) civil society organization representatives (2), nonactivist observers such as journalists and political strategists (2), public health officials (3), healthcare workers (2), academics (5; 3 in toxicology and environmental health, 2 in dental and public health). AB knew many participants from when the O’Brien Institute for Public Health was invited by the city council to prepare a knowledge synthesis of the data on community water fluoridation in 2019. Participants knew AB sought to facilitate dialogue without taking a public position on the debate itself; AB co-founded the UCalgary Pluralism Initiative and seeks to foster shared understanding across sectoral, disciplinary, and identity differences towards greater equity, inclusivity, and innovation. AB checked with members continuously throughout the interviews to ensure comprehension. Field notes were taken to document interviewer observations during the interviews. Interviews were audio-recorded and transcribed verbatim using Rev.com. Transcripts were checked by the researchers for accuracy. Participants were invited to check their transcript for feedback and only minor language clarifications were requested, and participants received a summary of findings which was met with a general appreciation concerning the synthesis and diversity of voices represented.

### 2.4. Interpretation

Transcripts were coded using NVivo by graduate student CSP, reviewed by principal investigator AB, and subsequently interpreted by CSP (public health graduate student, and woman), JPF (Track Two diplomacy expert, woman), PJ (Track Two diplomacy expert, man). AB (principal investigator, public policy expert, man) employed an inductive, thematic analysis to identify prominent themes within the data. To extract insights from our study data, we began with an open coding scheme [17]. This involved identifying themes that were relevant to the research question(s) and based on a review of the literature in the Track Two diplomacy field. These preliminary themes were then further refined into analytical categories to guide a second round of coding [17]. During this phase, our focus shifted from the specifics of our interview data to the conceptual and structural relationships between the categories and codes. Clusters of related concepts and categories were identified. Finally, we determined whether they could be further subdivided into dimensions or broader constructs. Through this iterative process of analysis, some themes emerged as more salient while others lost their initial significance. The results of this interpretation follow. Table 1 summarises the contextual factors that surfaced during conflict mapping about the CWF conflict in Calgary, Alberta. Further, in Table 2 we summarise the would-be model design features of a Track Two process as described to us by our participants.

## 3. Results

### 3.1. Contextual Factors of the CWF Debate

#### 3.1.1. Issue

##### Binary Issue

Participants told us that the CWF issue has a binary outcome—either the water is fluoridated or not—which positions all debate along a yay vs. nay axis that leads to entrenched positional advocacy. Many participants instead favoured reframing the issue to a higher-level topic that would position both pro- and anti-CWF advocates on the same side of the issue: e.g., child health. While within any discourse about child health, community water fluoridation would still emerge as a disagreement, the disagreement would emerge under conditions explicitly created to foster an orientation to common purpose: child health. Instead of nurturing entrenched positions, the structures would unite conflicting parties to a shared goal. This could hypothetically make joint problem-solving easier; by shifting from wants to deeper needs, the group could end with a larger coalition of like-minded participants to advocate for a more complete solution with greater impact.

*“So, what is the goal? I’m sure we could find agreement, even across the most intractable individuals. I think the goal is good dental health. And then what are the steps that we need to take as a society to help us achieve that goal?”*

—Interview 5

*“The conversation from public health is focused on fluoridation.... but it’s much broader than fluoridation. And yet it stays focused on that one thing which is the most polarizing thing. And so, I think we really missed the whole heart of the matter and that’s where I think we failed as leaders.”*

—Interview 5

##### Disciplinary Silos

Participants told us that data do not always give a clear answer when comparing results across disciplines; nuanced findings about risks and benefits can be difficult to compare across disciplines. Academics seem to privilege the arguments and frames of reference from within their own discipline. And when diverse disciplines do engage, they cannot find common ground on a collectively shaped path forward because they do not speak the same epistemic language—often shaped by discourse flavoured by non-academic, advocacy-grounded tones.

##### Failed Public Engagement

We were told that government’s public engagement on issues like CWF is superficial if not performative; abdicating a responsibility to facilitate and foster productive, constructive, and nuanced discourse. Modern politics casts a long shadow that influences how elected officials govern and what they expect of city administration; each failing to foster shared understanding, especially in a way that would connect members of the public, policymakers, and scientists.

#### 3.1.2. Societal

##### Populism

Participants described that our political systems have devolved into permanent political competition that has fuelled populism and our inability to dialogue with any objective common reality. In this context, we were told that political actors distort science towards their political goals, which in turn devalues scientific expertise. As populist tendencies surge, some participants suggested that when constituents do not understand or agree with the science, the ethics of political representation seems to increasingly come into conflict with the ethics of political leadership: do you follow your constituents, or do you follow the science?

*“There’s two challenges that we currently face. One is the death of expertise—that I have an opinion and my opinion is the same as your opinion and concurrent with the second is the rise in populism. It is really challenging to make these decisions.”*

—Interview 21

*“The project of Western Democracy was set up with the idea that we would have periodic competition with ideas for the right to govern, and… what’s happened is that we’ve become so skilled in that competition, that it’s become the canary that’s eaten the cat, and now instead of competing for the right to govern, the governing is in active constant competition, and that is an existential threat to the entire makeup of our society.”*

—Interview 4

*“Are you making a decision that’s rooted in the data, in the science and the ethics of what you’re supposed to do or are you doing it because you’re worried about getting re-elected—and that’s when your ethics goes right out the door.”*

—Interview 1

*“You are rewarded as a politician for doing what is popular. What is popular is not always right.”*

—Interview 21

##### Lack of Disposition to Dialogue

Participants told us that decisionmakers do not appear prepared to dialogue; they start from fixed positions and engage in debate rather than dialogue. We were told that this extends to all players in the conflict, from both proponents and opponents of community water fluoridation, who *“both look at the same study but arrive at vastly different conclusions. And that’s an example of confirmation bias—seeing, and, and being drawn to the evidence that supports your deeply held beliefs.”* (Interview 7). Yet, despite that acknowledgement, conflicting parties do not always seem interested to understand the other’s perspective. Members of the public were described as similarly polarized and not predisposed to dialogue: *“they just wanted to tell me how I should think and trying to have a dialogue with them was next to impossible, because they would come in and say, ‘what side are you on?’”* (Interview 1). We were told that the media also plays a role in hampering dialogue by forcing political candidates into binary positions without considering nuanced perspectives about how the issue is framed or what new data might change their minds. Furthermore, to protect themselves from being caught in political firestorms, we were told that many bodies that do research synthesis are hesitant to release controversial findings that erode open and timely knowledge sharing, thus further limiting any commitment to dialogue.

#### 3.1.3. Individual

##### Adverse Impact of Conflict

Many academics that we interviewed described being attacked with *“awful stuff”* (Interview 16) in the public arena and by other academics. Some academic voices expressed feeling marginalized in the academic community when their data or their interpretation put them outside of mainstream thought. Further, students and junior faculty were attacked and *“vilified”* (Interview 16); it had a particularly negative effect on their wellness and ability to function professionally. However, clinicians interviewed seemed less affected by the discord.

*“And so, [period of time] ago, I would have told you, “Diversity is strength,” but right now, being the different one, being the diverse one, has taken a serious toll, personally and workwise …. I’m not gonna lie.”*

—Interview 19

*“I’m not scared of it. And like I said, not worried about my reputation. But for younger people who are involved in this, this is what really has been upsetting to me.”*

—Interview 18

*“Things that happen that I don’t agree or, uh, makes me a little upset or frustrated but no, it’s just the nature of the job. (Laughs). It happens everywhere, and at the personal level, I don’t see anything.”*

—Interview 8

##### Extreme and Rigid Positions

Both opponents and proponents were described as rigid, despite both parties self-identifying as openminded:

*“Both sides often think that that they’re open to persuasion and that they’re open—but in practice I have found that they’re not amenable because they’re only willing to trade with others who are on the same level of discourse as them. And this problem happens a lot.”*

—Interview 21

Participants attributed some rigidity to professional inertia linked to historical positions:

*“Dental health promotion has been recommending water fluoridation for decades. And to [potentially] all of a sudden reverse course is just one of those seminal moments that every professional must feel incredible anxiety about. Because this is something that they’ve been telling the community for years.”*

—Interview 16

Further, when scientists are seen to be closed to dissenting views, we were told that they are perceived to be muting their academic peers:

*“Just asking questions and trying to figure out the balance of evidence—classic systematic review type questions like: here are some doubts—what should the future look like in terms of asking the right questions? You can’t do that with [this issue], right.”*

—Interview 13

However, participants explained that simple signals of openness allowed for discussion and relieved a need to immediately engage in positional advocacy.

*“I think the perception was when we entered the room that there was already a position, and when it was clear that this was more neutral and maybe they had anticipated, there was a willingness to engage in the conversation.”*

—Interview 3

One participant stated that scientists do not necessarily share positions with the advocates who cite and promote their findings—but that they are reflexively seen as allies or extensions of one another despite never choosing to be allied. This complicates scientific dialogue and in turn influences policy advocacy.

*“A community of anti-fluoridation folks have been beating this drum against water fluoridation, but some of it on pretty shoddy science which stoked passions for decades and created these polarized communities.”*

—Interview 16

Participants frequently noted that community members were resistant to changing their positions (regardless of the position itself):

*“I’m struck right now by the amount of people that cannot be moved positionally. I’m stunned, I don’t get it.”*

—Interview 20

##### Issue Fatigue and Apathy

We were told that decisionmakers want a decision now—even if wrong—because they are generally fatigued but also because they are particularly tired of this issue.

*“I think with people like me, you’ve reached a point of fatigue. Like I, I’m so sick and tired of doing the same thing over and over again, and that’s what we do. We will do this all over again.”*

—Interview 1

##### Lack of Humility

Leaders do not demonstrate the necessary humility to learn and operate in a manner that leads to the best solutions.

*“You have to have leadership that’s interested in practicing some humility, to not always have to win and understand that sometimes losing on your idea actually results in a better solution, but that’s not what we have. I would say globally, we do not have political systems that operate on that level. Everything’s a show of force.”*

—Interview 1

We were told that scientists and decisionmakers do not ask questions and assume more than they know, which adversely impacts their understanding of the data that subsequently informs their positions or votes.

*“A flaw in reasoning is the fact that we tend to think we know more than we do. I, myself have found that when I say, “Oh, look, the literature shows that it saves 0.5 of the tooth surface” and I’ll say, “Oh that looks like a very small effect”. And then, it’s my colleague who is the dentist will correct my thinking and say, “Actually, that’s a large effect”. And explain it to me in a way that I didn’t think of at a population level and how maybe one cavity then is predictive of other cavities over the lifespan. So, I think humans are just motivated to pretend that they know more than they actually do. And we’re not aware of our limitations. It’s not intentional; it’s just that we are not aware of what we don’t know the gaps are in our knowledge.”*

—Interview 7

#### 3.1.4. Policy-Making

##### Perceived Lack of Leadership

Due to their demanding schedules, participants explained that councillors preferred not to learn new information if a vote appears to be heading in a particular direction, as the outcome is already fixed.

*“Procedurally, you know that if you have eight votes, you don’t have to listen to anybody else. It’s just easy. And so, do you want to make quick, easy decisions as a politician? A hundred percent you do because it’s easy.”*

—Interview 1

We were told that rather than government embodying a learning system grounded in humble servant leadership, that councillors behave with bravado, winner-take-all mentality even if that hurts the public by not getting us to the best possible solution.

*“Now that Notice of Motion has become a tool to say, I have 10 people who signed onto this. So, you know what? Screw you guys. I don’t care that you don’t like this. I’ve already got my 10 votes. Let’s go. That’s how we do things. It is very much bravado. It is very much chest thumping, it’s performative. It is theatre at its best. It’s not good decision-making.”*

—Interview 1

##### Lack of Forum to Dialogue

Participants expressed that there is not a forum for dialogue—that city bylaws do not allow for meaningful dialogue outside of council chambers, which often get reduced to theatre because of the council’s public nature. There is no clear venue or method to tackle complex issues where councillors can gather, understand, and interpret the facts over time while in constant dialogue with each other, experts, and the public.

*“They say technically the lawyers have advised that if eight of us in Niagara Falls or wherever the hell we happen to be, decide to get together at a bar and say that everybody’s gotta wear socks in the city tomorrow, we could pass a bylaw that everybody has to wear [redacted] socks. Which is ridiculous.”*

—Interview 18

*“It’s systemic; the inability to make a decision using a proper tool or method is what gets in our way.”*

—Interview 1

Some participants told us that modern politics focuses on the politician’s positioning in the public eye towards re-electability, which detracts from a fact-based, public-good-oriented discussion or decision-making.

*“You will never have a durable political decision because that’s not what we do. We don’t make decisions based on facts. We make decisions based on going through this whole process of, if I do this, what will happen? If I do that what will happen? If I just wait and see how the others are gonna vote, does that put me in a better position? That’s what politicians do.”*

—Interview 1

Participants expressed that the CWF issue exemplifies a need for a forum to dialogue across orders of government because the CWF issue requires expertise, possibly funding, and operational facility control that cross government jurisdictions—but that the *“layers of decision-making complexity…are not aligned in most Canadian jurisdictions in terms of the levels of government”* (Interview 10).

With a plebiscite forthcoming at the time of this study, elected officials expressed frustration that the process will never end and no matter the result of the plebiscite, the losing side will simply say, *“‘Oh, I think we need to review it,’ and I’m just concerned that we’ll just continue to drag it out”* (Interview 2).

##### Polarization and Silos

Participants told us that they are constantly being pushed into polarized, siloed positions. Participants said that the nature of public discourse prevents leaders in any sector from asking questions because merely asking for more information or asking thoughtful questions often leads the public to jump to conclusions about which side you are on, which prevents dialogue. We were told that the current state of political discourse is losing the public’s trust in our collective ability to dialogue on a foundation of shared truths to solve problems.

*“You can’t ask a question without immediately trying to put you into one side or the other, right?”*

—Interview 13

*“The conversation has been framed as binary… either you believe in it or you don’t, it’s good or it’s bad, it’s right or it’s wrong, you do it or you don’t, there’s, there’s been very little space in the middle for what else it could look like.”*

—Interview 3

### 3.2. Designing a Track Two Process

Participants recognized the inherent personal and societal tensions embroiled in the CWF debate. Participants favoured trialling a Track Two process on the CWF issue. The general objectives of such a process would be to determine if a scope exists to introduce a “problem solving” dimension to the discussion over the issue, as opposed to a bargaining one, and also to observe if the deeply ingrained narratives that various players hold could be discussed in ways which were not threatening.

#### 3.2.1. General Structure

##### Reframe to a Nonbinary Issue

Participants felt that the problem must first be defined at a level that everyone can come to the table at with common purpose; the conversation should not be centred on a single binary choice.

*“If we can have a conversation about how that entire thing could look, like thinking of this as children’s health.... I think that as long...as we could get everyone on the same page of figuring out what that [umbrella topic] is, then for sure I think the conversations within it would be much more productive.”*

—Interview 21

##### Need for a Skilled Facilitator and a Deliberate Process

Participants thought that facilitated discourse could lead to progress if it attempted to foster a shared understanding both at the table and away from the table.

*“You need a thoughtfully designed agenda, so that it’s very clear what, what they’re there to discuss. There may need to be a, a kind of caucusing or pre-conversation before bringing anybody together. And probably throughout—a kind of facilitation—going apart, coming together, going apart, coming together and, and actively working with the groups in the apart phase as well.”*

—Interview 3

The facilitator should first focus on helping participants to identify their “*shared goals” and then to “acknowledge what they know, what they don’t know, and to uncover some assumptions...and where those beliefs came from*” (Interview 7). Without perhaps knowing it, this interviewee was talking about the shift from bargaining to problem-solving.

Participants expressed the need for the facilitator to be skilled and have the necessary attributes to identify and bring diverse voices together, creating safe spaces for dialogue.

*“The facilitator sets the tone around participation in wanting to hear from and seek out different voices…”*

—Interview 20

*“Throughout the meeting, there needs to be a commitment from people at the table that we are going to treat all members with dignity and respect and provide a safe space for people to be vulnerable and listen.”*

—Interview 24

##### Begin with Academics

As for those who would take part in these discussions, participants maintained that scientists first need to come together to find common ground across their epistemic conflicts before opening dialogues to other conflict players.

*“I would feel more comfortable with academics first…it’s, it’s an added layer of complexity to bring the public who may not have the training to understand what we’re saying. I mean, if we speak a common language and we don’t understand each other anyway, it’s complex to bring in somebody who doesn’t.”*

—Interview 19

However, it remains unclear as to whether it is best to bring together people who are very close to the science, perhaps even the original authors themselves, or to focus on those who understand the disciplinary contexts but who are not scientists themselves.

*“I have mixed feelings about people who are too close to the science.”*

—Interview 7

*“As an academic in that conversation, regardless of how I’m presenting myself or to what extent my identity is disclosed, I still will want to put my best foot forward and have at least a more open conversation than just, oh, I’m gonna dismiss everything you’re saying or just stick in my heels and refuse to listen.”*

—Interview 13

Participants encouraged involving *“normal people”* in dialogues to exert a moderating force on the discussion because *“that space, that dialogue is different than the one between the kind of two extreme groups”* (Interview 3).

A popular moderating approach was to include important non-academic stakeholders in discussions, such as parents and families. Specifically, participants wondered whether facilitating dialogues among scientists ought to additionally include policymakers and especially members of the public—like parents and families—hoping that it might exert a moderating influence on the debates to foster nuanced conversation.

Our participants considered inviting ”outsiders” fluent in academic language, or perhaps senior and/or retired scientists, to further moderate the discussion.

*“You need some scientists who are senior. Senior as being unbiased who would take enough interest or been recruited to essentially be impartial by conveners of the discussion.”*

—Interview 16

Further, there must be balance between peoples’ biases rather than trying to find an equal mix of participants from two conflicting sides.

*“…we wanna have a balance of bias, not balance of conflict.”*

—Interview 6

Eventual future steps need to take the discussion nationally and stay focused on the originally charted shared goal of, for example, child oral health.

*“At some point, all of that needs to be brought together in some kind of a national strategy, uh, federal strategy, to protect children from tooth decay. And, and at some point, we have to broaden that out beyond fluoride because focusing solely on fluoride is just idiotic.”*

—Interview 6

##### Generate Bundles of Solutions

Participants favoured dialogue that could produce a bundle of solutions. The goal should not be to reach a single solution but to produce a suite of possible paths or approaches that can help tackle the problem in different ways without falling into further yay/nay binary traps, allowing consensus to emerge on paths rather than positions, and for thoughtful dialogue to persist.

*“With most complex problems, there is not a single bullet policy intervention that’s gonna solve it. So, for us to believe (in) that one single policy intervention is problematic. It is the multitude of voices around the table that bring different options forward, thinking about how to collectively get to that outcome, where we can bring multiple solutions forward that would work in concert with one another, not compete with one another.”*

—Interview 20

##### Hold Multiple Tracks of Conversations

In armed conflicts where Track Two is traditionally used, the participants convey lessons from the discussions to their respective parties through a process known as transfer [18,19]. However, in the public health setting, there are not necessarily discrete parties—rather, informal affinity groups spread across wide geographies. In this more discrete context, participants encouraged multiple parallel conversations to be facilitated to allow for a rich societal-level discussion that would transform the discussion across society, allowing for a continuous and facilitated flow and collision of ideas and perspectives through the process.

*“…hold a series of these discourses so the transfer occurs naturally because you have a multiple, um, conversations that may all each go different directions, but along the way, there’s better understanding of the issues than each other.”*

—Interview 17

Participants proposed connecting these initial conversations between academics to the ongoing independent dialogues with policymakers and community. Further, these dialogues should not be single and linear, but continuous, *“multi-stage process[es], [and] also multi-pronged so there are discussions happening in parallel”* (Interview 13). Parallel dialogues could include *“parents and policymakers and toxicologists and public health officials and dental experts”* (Interview 6) to discuss specific issues. Furthermore, those different tracks and processes need to be in conversation with each other: *“…so I do like the idea that there’s an integration of the tracks. I also think it’s really important for all three of those tracks to be talking, to understand the different perspectives”* (Interview 20).

#### 3.2.2. First Theory of Change in CWF

In Track Two diplomacy, the “Theory of Change” concept is pivotal; it acts as a conceptual framework that defines the underlying causes of the conflict which practitioners take into account in designing an intervention comprising tactics and goals most appropriate to the situation. These theories allow for effective evaluation of dialogue outcomes against the motivating assumptions [14] (pp. 54–61).

Participants proposed the following theories of change for the community water fluoridation issue:

##### Changing Personal Attitudes and Relationships

The breakdown of trust and relationships amongst differing viewpoint advocates was deemed the primary cause of this conflict’s intractability. Participants emphasized *“trust building”* (Interview 21), *“humanizing”* (Interview 19), and a desire for *“less churn, less vitriol, [and] less problematic dialogue”* (Interview 10). Participants wished to move away from oppositional debate and towards constructive dialogue and shared understanding (Interview 11). To get there, “*there has to be an acceptance of and a willingness to say there are different perspectives”* (Interview 20). Further, with the right people in the room, *“the more time you spend with them, I think the more likely you are to work together”* (Interview 21). *“It’s difficult to hate people when you get to know them”* (Interview 19). An established theory in Track Two diplomacy is “intergroup contact theory.” This theory posits that initiating discussions to dismantle stereotypes and suspicions can be a useful initial step in conflict resolution [14].

##### Changing Culture

Another potential theory of change which may be relevant to this case concerns the political and social culture within which this dispute exists. Our participants noted that societal norms and structures seem to encourage us to *“exist in these little bubbles and silos where we take in information and we make up our mind, and then we come ready to debate and that’s not dialogue”* (Interview 1). This problem applies to proponents and opponents of CWF, but also across business units where policymakers *must “build relationships with different departments, different organizations, and in creating those relationships we have an ability to see others’ perspectives and get a differing understanding”* (Interview 14).

Here, the “community relations” theory of change suggested by Ross could be applicable. This theory of change suggests that the emergence of groups wherein participants in the conflict are sorted into already conflicting groups has the effect of further polarizing the participants and deepening the distrust they feel towards each other. In this situation, activities and dialogue are aimed at breaking down the “group” dynamic of the conflict in a manner that aims to bring out the differences within these groups—thereby demonstrating that these groups are not monolithic. By demonstrating diversity of views within any group, participants are less likely to see an entire group as singular, but as nuanced and varied, like in their own group. It is expected that this added texture will humanise the other side and open new possibilities to make the conflict less intractable [20].

Two specific cultural dimensions emerged as critical: gender norms and governance structures.

Gender norms:

*“I mean, women are typically the ones who look after and care for kids in most different societies, but it is the men who make the decisions on whether to allow that to happen or not in some cultures. And so, how do you hear the voice and concerns of the mom that’s raising the child and what they see is happening and the father who is ultimately determining whether the child gets [intervention] or not.”*

—Interview 2

Governance:

*“…reality is that we need an epochal shift in our governance structure to reflect our current reality, which is 180 degrees different from the governance environment when we first set things up.”*

—Interview 4

#### 3.2.3. Evaluation

One of the key areas in the study of Track Two diplomacy has been that of measuring and evaluating the results and the impact of such dialogues. Put simply: how do we know that such dialogues have had an impact and what has that impact been? Measuring the diffusion of ideas and changed attitudes through a complex and evolving society is not an exact science. Notwithstanding this challenge, participants in the interviews described success in terms of whether people felt heard and whether shared values were made manifest; if the group’s objectives were met (whatever they were); whether those who are not elite were included; if the plan was ultimately adopted (grant accepted, policy implemented); and others contended that effectiveness could not be assessed until years later. In this way, the findings are broadly consistent with the field’s key assumptions and findings [21,22].

*“Wouldn’t the success be the, that orders of government adopt the measures that are recommended?”*

—Interview 5

*“Say we came up with a [Track Two process] and just start using fluoride as an example of how to solve that problem and then how to use the way we solve that problem to develop a framework of how society interacts with science.”*

—Interview 15

*“Do I feel like I’m being heard? Do I feel that we’re working towards whatever shared goals that we originally [set].”*

—Interview 21

*“That it’s not just talking heads or that people who are there to represent specific scientific expertise or a health agency, that it was … mothers, who are gonna be the ones impacted by the decisions made in this group.”*

—Interview 6

*“You have to look back at the process several years later to see if you got it right.”*

—Interview 18

#### 3.2.4. Anticipated Benefits of such a Dialogue

##### Generative

Almost all participants felt facilitated discourse would be a worthwhile approach to help resolve intractable policy conflicts like CWF. At present, there is not a clear neutral forum *“to learn, to seek understanding, and to be open-minded to difference”* (Interview 14).

*“It has to be part of the future because I really am concerned that if we don’t figure this out or figure something out and try something that I just fear for the future of democracy.”*

—Interview 21

*“It feels like these intractable conflicts just go in circles in some way. And so disrupting that and kind of breaking it up and bringing in other people, I think that’s exactly what we need—getting people to think through something rather than default to just a position.”*

—Interview 11

One participant, previously skeptical, was less dubious after the interview, stating, “*I gotta say, you moved me a little bit during the conversation from kind of thinking that, gosh, this is not something that could ever work, you know, uh, to it’s possible*” (Interview 13).

A concern raised, which reinforced the preference to start with academics, was that it might be hard to generate interest from non-academics to participate in this exercise: *“I’m not fully persuaded that you could get enough people on board to, to do it outside of the scholarly community. I see a lot more scope for it [in academia] but in other settings, I feel like it would, it would be hard to get the public engagement”* (Interview 17).

##### Steward Common Purpose

We were told that by focusing the Track Two processes on higher levels that reframe the dialogue to a level that everyone can agree on, using terms that we all understand, we can begin to depoliticize the otherwise intractable discussion and come at it from new ways and with new trust.

The Track Two problem solving approach has potential to help us *“find common purpose, especially across silos or across ideologies, and across differences”* (Interview 1) by helping us learn from different perspectives and to depoliticize decision-making. We were encouraged to change the language from ”conflict” and “facilitated discourse” and towards notions of *“how to depoliticize decision-making”* (Interview 1), and to acquire a marketing team to strategize better ways to describe this work that *“makes people feel like they will be better and stronger as public service by doing this...it’s not that [these terms are] off-putting; it’s just that people don’t get [these terms]”* (Interview 1).

*“I think if you’d started with the outcome around healthy teeth and a full health perspective, a physical health perspective, and you focused on the outcome that you were seeking as opposed to the intervention and whether or not you support the intervention, that might be an entirely different dialogue.”*

—Interview 20

##### Cautious Optimism

The problem with this approach, according to some participants, was that the motivations to participate in discourses that arise through armed conflict are entirely different than in public health contexts.

*“In conflict situations war, it’s been successful, but both parties have no choice, but to come the table because they can’t go on in, in a serious conflict, they can’t go on like that. But in these discussions of policy and health, how do we make it so people have a vested interest to have that conversation and to be open to those ideas and move from their position.”*

—Interview 9

Further, unhealthy contact can reinforce existing positions and prejudices; so, if the sessions are not effectively constructed and facilitated, they could do more harm than good.

*“The more we can do on the human side, I think the better. But the more that fails on the human side, the more we hate each other too. So, (laughs) you know, when you try and reconcile with someone on a personal level, and if, if it doesn’t work out, it almost reinforces that people are unreasonable. It never works out.”*

—Interview 21

*“In an international colonized environment and someone with very strong colonial approaches, then there are, there’s an ethical factor there in how you’ve set the table for different people to participate to come at, to come at a challenge. And then, there would be different factors around gender, around inclusion.”*

—Interview 19

## 4. Discussion

This study aimed to examine whether and how facilitated discourse (Track Two diplomacy) might help transform the intractable CWF policy issue in Calgary. Participants expressed cautious optimism that facilitated discourse could help reframe the issue from a binary (win/lose) positional debate to a more constructive conversation about child health. The policy-making milieu features leaders and processes characterized by bravado, short-term thinking, and a winner-take-all mentality that do not make way to explore complex topics, to privilege relationality, nor heal and grow as a community on contentious policy issues. Participants described the populist tectonic plates shifting underneath our current society, which are giving way to multiply held truths, rigidity, and disrespect for science—culminating in a more complicated scientific discourse arising from, at times, vicious social media and activist attacks that leave us unable or unwilling to dialogue across differences. Furthermore, many in the silent majority do not see themselves in the vitriolic discourse and so retreat, thereby creating more space for extremists to shape tone and policy, which seeps into scholarly discourse.

Participants suggested that a Track Two process might start by convening conflicting academics from diverse disciplines, first to humanise each other and reduce the temperature of the conflict between them as an initial theory of change before moving on to jointly assess the existing science and design a bundle of potential paths forward, perhaps including joint studies to reconcile these differences.

Participants opined that inviting “normal people” into those dialogues could serve as a moderating force on those discussions, and concurrently holding many parallel dialogues among multiple diverse stakeholders that might simultaneously educate the public about the issue from different perspectives, while role-modelling civil discourse in pursuit of truth.

Track Two diplomacy offers an appropriate methodology by helping conflicting parties move from positional, adversarial, and bargaining towards collaborative dialogue grounded in emergent trust. Notably, the mechanics of facilitation are not arbitrary; they are carefully considered based on a predetermined theory of change and grounded in a robust understanding of the conflict and its context and players [20,23]. The application of Track Two methods to public health policy conflicts is informed by scholarship in political science and theories surrounding participatory governance, which includes civil society in political decision-making [24].

Track Two diplomacy has evolved from what was traditionally a process among elites to one that strives to be more inclusive of civil society, such as women’s organizations, minority groups, and business and religious leaders, in peace deliberations [25,26]. Having more agents complicates the process and makes agreements more difficult to attain, but there is evidence the achieved outcomes are more durable [27]. While low-level consultation such as town halls, demonstrations, and petitions are commonplace [24], scholars are increasingly pointing to Track Two diplomacy as a more active, facilitated process to overcome intractable conflicts, particularly in an era of declining public trust in government and in the validity of scientific ”facts” [28]. This study contributes an early application of Track Two beyond the international relations conflict resolution field, amidst an increasing curiosity to apply the Track Two process in other contexts, such as public health [1,11,29].

Track Two diplomacy, a mechanism grounded in social psychology, has the potential to bridge both the rational and emotional elements that contribute to intractable conflicts. In its ideal application, facilitated dialogue would shift leaders and scientists away from positions of certainty to ones of humility, creating space for disagreement that does not end in defeat. Rather than being ”right” or ”wrong”, those involved in discussion ought to be pushed to think reflexively—perhaps the greatest feat possible in intractable debates. Further, creativity in conversation spurs ingenuity; for issues that have grown tired, such as CWF, new possibilities are required. People hold astonishing innovative potential; the question is how best to harness our inherent human creativity within traditional, policy-making spaces [30]. In this way, Track Two processes might help to shift our current policy-making milieu: introducing new ideas to the collective through alternative channels of communication and exploration. Possibilities for change and innovation with public health policy problems are, perhaps, just adjacent to the situation, we need only to look [30].

Towards greater innovation, Track Two facilitators might employ a variety of techniques: adversarial collaboration combines diverse perspectives and skills that could lead to better understanding of the science by all parties and even lead to better quality if not novel research to advance the field in service to child health [31]. Ritual dissent offers an approach to critique (dissent) and build (assent) ideas towards a stronger final product [32]. In an appropriately designed setting, either approach could lead to constructive collaboration.

Facilitated discourse might not be the panacea to all public health conflicts. Participants raised questions whether Track Two diplomacy would work as well in the field of public health as it does in situations of armed conflict. How do we create the necessary buy-in to sustain discussions over time, especially when elected officials change and public attention wanes in the face of a more imminent crisis? Finally, participants championed caution in bringing together diverse individuals; if the sessions are not effectively constructed and facilitated, we could do more harm than good by reinforcing existing prejudices and positions.

This study has limitations. Participant selection might have introduced bias since the committee comprised individuals willing to convene and find a path forward on an intractable conflict, and so are perhaps predisposed to having “moderates” in their networks; however, to move this issue forward requires convening disagreeing moderates who have the potential to chart a new path forward, so while this is a limitation to generalization, it remains an appropriate process to identify salient issues for a future Track Two process. The participants were educated and not representative of the general public. The interviews represent participants’ self-reported experiences and perceptions, which may be subject to recall bias or social desirability bias. The study relied on a limited number of interviews to gather data, rather than assessments at a massive city-wide scale and rather than direct observation of community, academic, and policy-making processes. The study used a cross-sectional design, which provides a snapshot of the participants’ perspectives at a single point in time. Although this case study focuses on community water fluoridation in Calgary, Canada, the issues raised seem applicable to a wide range of nonviolent public health conflicts. However, the nuance and depth of the issues raised are particular to community water fluoridation, which limits generalization to other social, geographic, or cultural contexts.

The most important future direction is to empirically assess whether the concept of Track Two diplomacy is effective in public health contexts, starting with the participant favoured approach here—to run a Track Two process amongst academics across disciplines while including “normal people” to moderate the discourse.

These issues would additionally benefit from marketing scholars and behavioural economics scholars to assess whether and how to encourage participation in such dialogues aimed at finding common purpose. Artificial intelligence scholars might consider how to embed the virtues of facilitated discourse in its exploding set of platforms and applications (whether search engines such as Google or ChatGPT, or social media platforms such as Twitter) to shape discourse at the point of initial public engagement with the topic rather than on the back-end after conflict has already stewed.

## 5. Conclusions

This study investigates the prospective application of Track Two diplomacy in tackling intractable public health policy disputes, using the community water fluoridation (CWF) policy issue in Calgary as an example. Our research indicates that transforming the binary (“for” or ”against” CWF) debate into a more comprehensive dialogue centred on child health could facilitate collaboration, promote innovation, and lead to long-lasting solutions in public health policy-making. Crucial to this transformation is the assembly of transdisciplinary academics who can jointly assess and address the problem by harmonizing divergent epistemic perspectives. Involving diverse public stakeholders in this facilitated discourse can further foster an atmosphere of respect and pragmatism.

Track Two diplomacy offers established methodologies that uniquely equip stakeholders to navigate the complex interplay of scientific evidence and policy discourse, which is often mired in entrenched positions, conflicting ”truths” and skepticism towards science. Future research should evaluate the broader applicability and effectiveness of Track Two diplomacy within public health contexts and identify best practices for engaging a diverse array of stakeholders in constructive, inclusive dialogues.

## Figures and Tables

**Table 1:** Conflict map of issues in the intractable community water fluoridation conflict in Calgary, Canada.

Issue	Societal	Individual	Policy-Making
Binary Issue	Populism	Adverse Impact Of Conflict	Perceived Lack Of Leadership
Disciplinary Silos	Lack of Disposition To Dialogue	Extreme and Rigid Positions	Lack of Forum To Dialogue
Failed Public Engagement		Issue Fatigue and Apathy Lack of Humility	Polarisation and Silos

**Table 2:** Design features for a Track Two process for the community water fluoridation conflict in Calgary, Canada.

General Structure	First Theory of Change	Evaluation	Anticipated Benefits
Reframe to Nonbinary Issue	Changing Personal Attitudes and Relationships	Driven by Theory of Change	Generative
Need for a Skilled Facilitator and a Deliberate Process	Changing Culture		Steward Common Purpose
Begin with Academics			Cautious Optimism
Generate Bundles of solutions			
Hold Multiple Tracks			

## Data Availability

We are happy to answer questions about the qualitative data while protecting participant anonymity, as described by the University of Calgary’s Conjoint Health Ethics Research Board.
